# Coach2Move: a person-centered physical therapy approach to pain-related functional decline in older adults

**DOI:** 10.3389/fpain.2026.1979758

**Published:** 2026-10-14

**Authors:** Ward Heij, Anne Thackeray

**Affiliations:** Department of Physical Therapy and Athletic Training, University of Utah, Salt Lake City, UT, United States

**Keywords:** geriatric pain, mobility, motivational interviewing, person-centered care, physical activity, physical therapy, self-management

## Abstract

Pain and physical inactivity are closely connected in older adults. Pain can restrict activity and participation, while inactivity contributes to deconditioning, disability, and persistent pain. Physical therapy is well positioned to interrupt this cycle, but care may insufficiently address factors shaping daily activity. In this Perspective, we describe Coach2Move, a person-centered approach aligned with the Age-Friendly Health Systems framework for older adults with mobility limitations and chronic conditions. Coach2Move operationalizes recommended geriatric physical therapy principles by integrating comprehensive assessment, clinical reasoning, motivational interviewing, shared decision-making, meaningful goals, and progress monitoring. Its contribution is the integration of these components into a defined process connecting impairment-level treatment with meaningful activity and self-management. Dutch randomized and pragmatic cluster-randomized trials found improvements in physical activity, functional mobility, and frailty with fewer sessions than usual physical therapy. We also describe adaptation to US outpatient care, including explicit content addressing pain-related fear, catastrophic interpretations, and beliefs that restrict activity. Coach2Move provides a structured method for integrating person-centered coaching with physical therapy for pain-related functional decline. Its pain-specific effectiveness and implementation in the United States remain to be established.

## Introduction

For many older adults, pain is not experienced as an isolated symptom. Pain can limit walking, household tasks, caregiving, and social participation, and even modest reductions in physical activity can accumulate into losses in strength, balance, and confidence (1). As activity narrows, dependence and reduced participation can follow. Clinical practice guidelines recommend nonpharmacologic strategies, with physical activity as a core treatment for chronic pain (2–4).

Age-Friendly Health Systems (AFHS) aim to improve geriatric care by aligning evidence-based practices with what matters to older adults and their family caregivers. The framework centers on four elements (the 4Ms): What Matters, Medication, Mentation, and Mobility; Multicomplexity has been proposed as a fifth M (5, 6). Together, these five domains emphasize an interdependent approach, recognizing how deficits in one can compound risk in others. Pain can influence all five components.

PTs are well positioned to address pain within this framework. Repeated visits create opportunities to support mobility, pain coping, behavior change, and self-management (7). US practice is heterogeneous, and many PTs, particularly those with geriatric expertise, already use person-centered goals, shared decision-making, behavioral strategies, and contextual assessment. Their consistent integration, documentation, and monitoring nevertheless vary. Coach2Move does not replace geriatric PT best practice; it structures these principles to connect physical capacity with meaningful daily activity.

This Perspective uses Coach2Move to illustrate how person-centered and age-friendly principles can be operationalized for older adults with pain-related functional decline. We describe its clinical logic and evidence, relationship to geriatric PT best practice, adaptation from the Netherlands to a US pain context, and implications for practice and implementation research.

## Pain-related functional decline is more than an impairment problem

Pain may aggravate during activity, but anticipated pain can be equally influential. Previous painful experiences, concern about tissue damage, or uncertainty about safety may prompt avoidance. Its short-term relief can reinforce inactivity, contributing to deconditioning and greater difficulty with daily tasks (9–11). Figure 1 illustrates how Coach2Move seeks to interrupt this cycle through meaningful goals, shared planning, monitoring, and adaptation aimed at sustained activity and participation.

**Figure 1:**
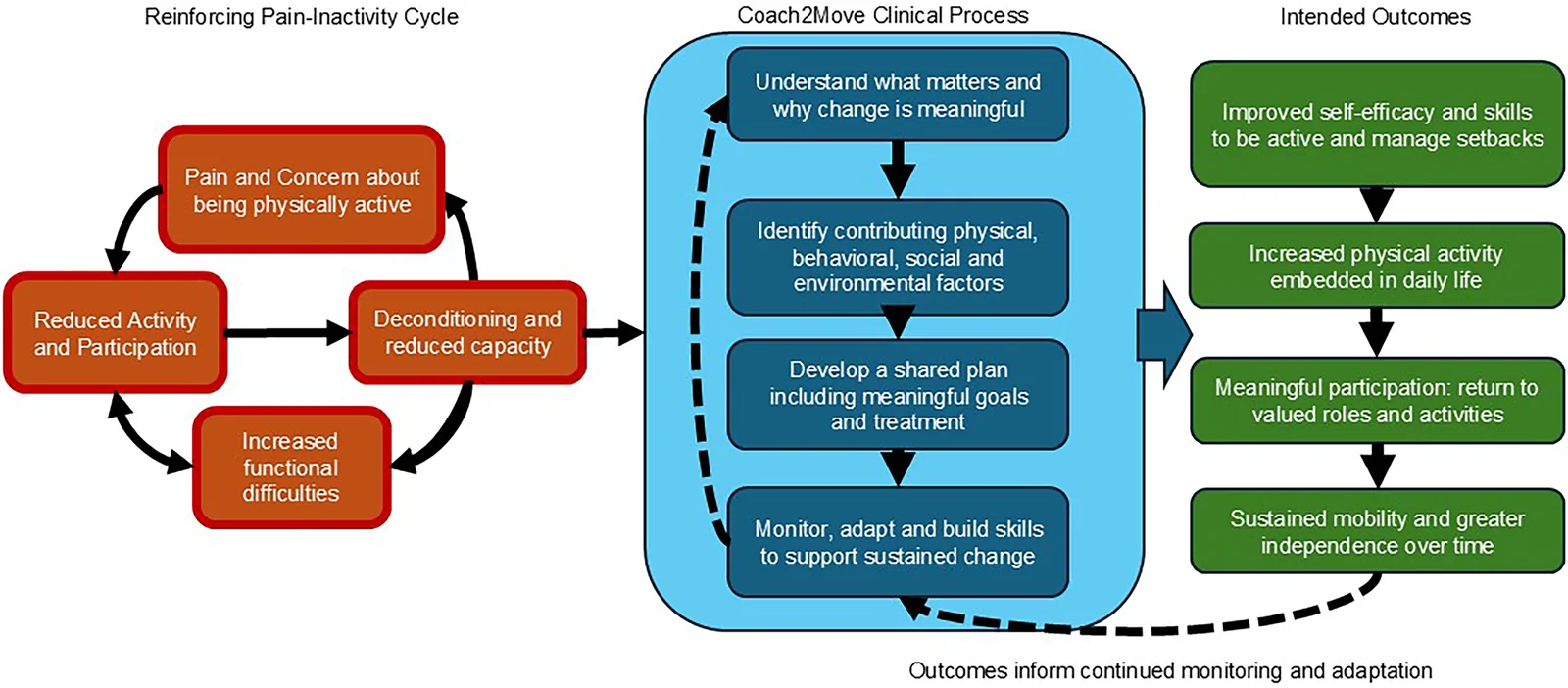
Interrupting the pain-inactivity cycle through Coach2Move. Pain and concern about being physically active can contribute to reduced activity and participation, deconditioning, and greater functional difficulty, thereby reinforcing the cycle. Coach2Move seeks to interrupt this cycle through an iterative clinical process in which the therapist explores what matters to the patient, identifies contributing physical, behavioral, social, and environmental factors, develops a shared plan, and supports sustained change through monitoring and adaptation. The intended outcomes are improved self-efficacy, increased physical activity, meaningful participation, and sustained mobility. Progress toward these outcomes informs continued monitoring and adaptation. Although pain may improve, the primary aim of the Coach2Move process is to support meaningful and sustainable activity rather than pain elimination alone.

Recognizing the behavioral and psychosocial factors that influence activity does not make the pain less real, nor does it imply that patients should simply push through it. It means that pain, beliefs, physical capacity, emotions, social circumstances, and behavior interact. Non-pharmacologic interventions that incorporate physical activity and psychological approaches can improve pain interference, physical performance, self-efficacy, and pain-related beliefs in older adults, although effects are generally modest and the supporting evidence varies in quality (12). The clinical challenge is therefore not to persuade every patient to exercise despite pain. It is to understand what the patient believes about pain and physical activity, identify what is physically and practically possible, and agree on a route back to meaningful activity.

Physical therapists may address these factors within usual care, but the consistent delivery of biopsychosocial and person-centered care can be affected by clinician knowledge and skills, available time, funding and service-delivery models, organizational resources, and referral options (13). These are multilevel implementation barriers rather than deficiencies that Coach2Move can resolve through the patient–therapist interaction alone. At the clinical level, Coach2Move structures assessment, shared reasoning, treatment planning, behavior-change support, and monitoring. Organizational and health-system barriers require complementary implementation strategies. Coaching does not replace person-centered geriatric PT or constitute a separate category of treatment. In Coach2Move, coaching refers to the systematic use of communication and behavior-change strategies across the episode of care to help patients identify personally meaningful goals, explore ambivalence, make informed choices, develop problem-solving and self-management skills, monitor progress, and sustain activity with decreasing reliance on the therapist. In addition to asking, “*Which intervention should I provide for this impairment?”*, the therapist also asks, “*What does this person want to be able to do, what is getting in the way, and what support would make change possible?”* This places clinical expertise within a broader clinical reasoning process rather than replacing impairment-focused assessment or treatment.

## Coach2Move in clinical practice

Coach2Move was developed in the Netherlands for community-dwelling adults aged 70 years and older with mobility limitations, physical inactivity, or risk of losing an active lifestyle (8). The approach combines comprehensive assessment with structured clinical reasoning and motivational strategies. These individual principles are not exclusive to Coach2Move and overlap substantially with competencies expected in contemporary geriatric physical therapy. Coach2Move is distinguished primarily by how these principles are configured and operationalized: a defined extended intake; hypothesis-oriented analysis centered on activity and participation; stratification of treatment intensity; explicit integration of motivational and self-management strategies; monitoring of activity outside the clinic; and a planned transition toward independence. Although treatment content remains individualized, the following six features define how these principles are organized within Coach2Move.

First, the therapist starts with an extended intake that explores why the patient seeks care and the personally meaningful goals underlying it. Rather than stopping at a goal such as increasing walking distance, the therapist asks where the patient wants to go, what they want to be able to do, and why it matters. The intake also examines health conditions, physical capability, and barriers and facilitators within the social and physical environment. The purpose is to understand how the mobility problem affects daily life and what meaningful improvement would look like from the patient's perspective.

Second, findings are organized through a hypothesis-oriented clinical reasoning process. PTs consider which factors are modifiable, which are likely to influence activity and participation, and which require referral or coordination with other professionals. In an older person with knee pain, quadriceps weakness may matter, but so may fear of falling, poor sleep, concern about worsening joint damage, a spouse who discourages activity, or the absence of an accessible place to walk.

Third, the patient and therapist make decisions together about goals and treatment. Goals are framed around meaningful activity rather than only impairment-level change. Range of motion or walking tolerance may remain relevant, but these become steps toward shopping independently, tending a garden, returning to church, or caring for a partner. The patient's priorities help determine which outcomes deserve attention and which tradeoffs are acceptable.

Fourth, the therapist uses motivational interviewing and related communication strategies to explore ambivalence and elicit the patient's own reasons for change. This is important because ambivalence is common and rational. An older adult can value independence while also fearing that increased activity will worsen pain. The therapist does not resolve that tension by arguing for exercise. Instead, the therapist listens for both sides, provides information with permission, and helps the patient consider a safe and personally acceptable next step.

Fifth, treatment emphasizes self-efficacy and self-management. Progress is monitored and discussed, setbacks are anticipated, and activities are practiced in the settings in which they ultimately must be sustained. PTs can also involve family members, friends, caregivers, or community resources to support the patient's goals. The intent is to reduce dependence on the therapist over time.

Finally, treatment intensity is stratified according to the patient's expected course and support needs. Coach2Move does not assume that more visits produce better outcomes. The number and focus of sessions are matched to condition severity, multicomplexity, and psychosocial needs, while the patient and PT maintain a shared expectation that gains need to continue outside formal care. This expectation is discussed early to support a planned transition toward self-management.

The proposed contribution of Coach2Move lies in the systematic integration of these components rather than in the novelty of any single component. Evidence for motivational interviewing alone in older adults remains limited, and evidence across adult populations suggests that its independent effect is difficult to separate from the broader intervention in which it is delivered (14, 15). Our own systematic review similarly found very low-certainty evidence for individual motivational strategies, while interventions combining several strategies showed a tendency toward greater effectiveness (16). Coach2Move therefore does not depend on one communication technique. It combines motivational interviewing, tailoring, shared decision-making, use of social context, and monitoring and feedback within a structured clinical process.

## Evidence from clinical trials and implementation

A pilot informed a randomized trial of 130 adults aged 70 years or older with mobility problems across 13 Dutch practices (8, 17). Usual physical therapy meant care without Coach2Move in participating practices, not a uniform model of PT. At six months, Coach2Move produced greater gains in moderate-intensity activity and reductions in frailty, with fewer sessions, lower health care costs, and a small gain in quality-adjusted life years.

A cluster-randomized stepped-wedge trial included 292 community-dwelling older adults across 16 practices (18). Differences favored Coach2Move for physical activity, functional mobility, and frailty at six months and were largely maintained at 12 months. Treatment sessions decreased, although anticipated cost savings were not observed.

The second trial took place during the COVID-19 pandemic, when treatment delivery and opportunities for community activity were substantially disrupted. Replication of the clinical findings under these conditions is encouraging, but it should not be overstated. Both effectiveness trials were conducted in the Netherlands. The first evaluated Coach2Move when delivered by geriatric PTs, whereas the subsequent pragmatic study expanded its delivery to PTs without geriatric specialization and used a multicomponent implementation strategy. These findings should not be interpreted as demonstrating that Coach2Move contains principles absent from geriatric PT best practice. Rather, the trials evaluated whether organizing these principles within the defined Coach2Move process improved outcomes relative to usual care in the participating practices. Both studies enrolled older adults with mobility problems rather than selecting specifically for chronic pain, so their findings do not establish Coach2Move as a pain treatment.

Process evaluation showed variation in adoption and fidelity (19). Education was combined with peer-assessment meetings, electronic health record changes, and reflection on clinical use. These were implementation strategies—not components of the patient–therapist interaction—designed to integrate Coach2Move into workflows. Because coaching may change familiar routines and views of professional expertise, a manual or one-time workshop is unlikely to be sufficient.

Taken together, the evidence supports two conclusions. Coach2Move can improve activity and function among older adults with mobility limitations, with fewer treatment sessions than usual care. The evidence remains context-bound: pain-specific effectiveness and implementation outside the Dutch health care system require further study.

## Adapting Coach2Move to a United States pain context

Clinical practice guidelines recommend physical activity and pain coping interventions as first-line, core treatments for chronic musculoskeletal pain (2–4). Coach2Move provides a strategy for implementing these recommendations. Transferring it to the United States required more than translating training materials because referral practices, professional specialization, reimbursement, visit patterns, and community resources differ. Researchers used Implementation Mapping and engaged researchers, physical therapists, patients, social workers with motivational interviewing expertise, and health system partners (20). The initial target population was adults aged 50 years and older receiving outpatient PT for chronic musculoskeletal conditions, including chronic low back pain, hip or knee osteoarthritis. These adaptations should not be interpreted as innovations beyond contemporary best-practice geriatric pain care. Their purpose was to make pain-related content explicit and teachable within Coach2Move and to adapt the program to the target population, workflows, and implementation context of US outpatient physical therapy.

Relative to the original Dutch Coach2Move program, the change in target population made chronic pain more prominent as both a reason for seeking care and a potential barrier to activity. The original program already required therapists to explore beliefs, expectations, confidence, and contextual barriers; therefore, these principles were retained rather than newly introduced. The US adaptation made pain assessment and management more explicit by adding training content addressing pain-related fear, catastrophic interpretations of pain, beliefs about harm, symptom-related uncertainty, and graded re-engagement in meaningful activity. These modules were intended to help therapists respond when patients interpreted pain during physical activity as evidence of harm, feared exacerbation, or felt unable to begin meaningful activity until pain had resolved. These additions represent adaptations to the original Coach2Move program, not claims of novelty relative to recommended geriatric pain-management practice.

Pain education is integrated into collaborative conversation rather than a standardized lecture. The therapist explores the patient's concerns, asks permission to share information, and links explanations to a valued activity. The aim is a shared, appropriately cautious understanding of symptoms that supports graded activity when indicated. Pain neuroscience education may improve pain, disability, and psychosocial outcomes for some people with chronic musculoskeletal pain, particularly with active treatment, but is not equally relevant or effective for all (21).

Distinguishing individualized, collaborative pain education from indiscriminate reassurance is particularly important in geriatric care. New or changing pain can signal injury, inflammatory disease, fracture, infection, malignancy, or another condition requiring medical evaluation (22, 23). A coaching approach should not dismiss symptoms or substitute reassurance for assessment. Self-management should also not shift responsibility onto older adults facing structural barriers such as unaffordable care, unsafe neighborhoods, inaccessible transportation, or limited social support.

Adaptation also raises a question of fidelity. If every component is changed to fit a new setting, the intervention can lose the features that made it effective; if nothing is changed, it may not fit the new population or care system. For Coach2Move, the intended functions should remain stable, whereas the specific methods and materials used to achieve them may be adapted to the setting. The intended functions include systematic attention to goals and context; shared clinical reasoning and decision-making; support for motivation, self-efficacy, and self-management; translation of clinical gains into daily activity; and a planned transition toward independence. Training formats, documentation tools, educational materials, visit schedules, and follow-up procedures may be adapted while preserving these functions.

## Implications for practice and health systems

Coach2Move suggests several practical changes for clinicians. First, activity and participation should be treated as primary outcomes rather than expected byproducts of reducing pain or correcting impairments. A patient may make meaningful progress even when pain persists, while pain reduction alone may not translate into greater activity or participation. Pain intensity still matters, but it should be considered alongside pain interference, mobility, confidence, meaningful goal attainment, and sustained activity. Evidence from other rehabilitation populations also shows that gains in physical capacity do not necessarily translate into greater activity (24, 25).

Second, clinicians should separate education from persuasion. When a patient is hesitant to move, more information is not always the missing ingredient. Clinicians need to understand what the patient fears and which outcomes are worth pursuing. Advice is more useful when it responds to those concerns within a collaborative plan, and motivational interviewing may help in this process (26, 27).

The 5Ms, already reflected in geriatric PT education and best practice, organize how Coach2Move addresses age-relevant concerns. It aligns most directly with What Matters and Mobility, while its assessment considers how Multicomplexity, Medication, and Mentation affect activity and participation (Table 1). Implementation requires organizational support beyond the patient–therapist interaction, including comprehensive assessment, documentation visible across the care team, referral pathways, and training with observed practice, feedback, and peer learning. Performance measures should extend beyond visit volume to meaningful goals, mobility, participation, and sustained physical activity.

**Table 1:** Application of the age-friendly health systems 5ms within Coach2Move.

Age-Friendly domain	Relationship to Coach2Move	Implications when applying Coach2Move
What Matters	The extended intake explores not only why the patient is seeking care, but also which activities and roles are personally meaningful and why improving mobility matters in the patient's daily life.	What Matters guides shared goal setting, treatment decisions, and the selection of outcomes used to evaluate progress.
Medication	Medication management is not a core component of Coach2Move. However, medication effects such as dizziness, fatigue, sedation, orthostatic symptoms, or inadequate pain control may restrict mobility and participation.	Physical therapists can recognize possible medication-related concerns, consider their effects when planning treatment, and communicate concerns to prescribers or pharmacists for review.
Mentation	Cognitive impairment, depression, and other changes in mentation may affect communication, safety, goal setting, and the patient's capacity to engage in treatment and self-management.	The extended intake may identify concerns that require adaptation of communication or treatment, involvement of a caregiver, or referral for further assessment. Coach2Move does not replace formal evaluation or management of cognitive and mental health conditions.
Mobility	Coach2Move assesses physical capacity, functional limitations, and personal and environmental factors that support or restrict activity. Treatment is directed toward improving participation in meaningful activities and sustaining physical activity beyond the treatment period.	The therapist combines appropriate physical treatment with coaching, graded activity, and self-management strategies tailored to the patient's goals and circumstances.
Multicomplexity	Understanding the patient's context is a core component of the Coach2Move intake. This includes assessing comorbid conditions and social determinants of health to consider how they affect the pain condition.	Contextual factors inform treatment priorities, adaptations, and the anticipated duration and frequency of care. The PT also identifies concerns that may require referral, interprofessional collaboration, or connection with health and social services.

This table uses the 5Ms to organize age-relevant considerations within Coach2Move. It does not imply that these considerations are unique to Coach2Move or absent from other high-quality geriatric physical therapy approaches.

## Future directions

Several questions remain. First, Coach2Move should be evaluated in populations selected for chronic pain and pain-related interference, using outcomes such as pain interference, self-efficacy, fear of movement, goal attainment, physical activity, and function. This would clarify whether the pain-focused adaptation adds value and for whom.

Second, implementation research is needed across different United States settings. Academic outpatient clinics, rural practices, safety-net systems, home health, and community-based services operate under different constraints and contexts. Research should examine reach, adoption, fidelity, adaptation, cost, and sustainability rather than focusing only on patient outcomes. It should also determine whether Coach2Move can be delivered equitably to people with limited health literacy, cognitive impairment, transportation barriers, or few community resources.

Third, future work should examine working mechanisms. Improvements may result from better clinical decisions, stronger therapeutic alliance, increased self-efficacy, more meaningful goals, changes in pain beliefs, greater activity, or combinations of these factors. Understanding these mechanisms would support more precise adaptation and help identify which elements must be preserved.

Finally, coaching should not be presented as universally brief or inexpensive. Some patients will need prolonged or episodic support, and fewer visits should be an outcome of appropriate care rather than a target imposed in advance. The purpose of stratification is to match care to need, not to ration it.

## Discussion

Pain-related functional decline in older adults is unlikely to be solved by impairment-focused treatment alone. Exercise, manual therapy, and other PT interventions can improve symptoms and capacity, but patients must ultimately translate those gains into activities performed without the therapist. That translation is behavioral, social, and contextual as well as physical.

Coach2Move structures the integration of clinical reasoning, meaningful goals, contextual assessment, behavior-change support, stratified care, and transition toward self-management. Dutch trials found improved physical activity, mobility, and frailty-related outcomes with fewer sessions. The US adaptation makes pain education and management more explicit, but its effectiveness and implementation remain unestablished.

Integrating coaching with treatment does not diminish the importance of careful assessment, diagnosis, symptom management, or appropriately dosed exercise. The distinction lies in how these clinical activities are connected to meaningful goals, self-management, and sustained activity beyond the clinic. Within Coach2Move, impairment-level interventions are therefore used as part of a broader process intended to help the person return to a meaningful and sustainable pattern of activity. For geriatric pain care, coaching could offer a practical nonpharmacologic direction that may complement broader efforts to reduce reliance on medication. It requires clinicians who can work with uncertainty and ambivalence, organizations that support longer-term outcomes over visit volume, and research that tests both effectiveness and implementation across diverse settings. The central question is not only whether physical therapy can reduce pain, but also how it can help older adults maintain or resume the activities and roles that pain has begun to restrict.

## Data Availability

Publicly available datasets were analyzed in this study. This data can be found here: https://repository.ubn.ru.nl/handle/2066/239796.
